# Transforming the language used in tuberculosis care

**DOI:** 10.1371/journal.pgph.0001657

**Published:** 2023-03-23

**Authors:** Beauty Umana, Jessica Vorstermans, Rhoda Lewa, Deliana Garcia, James Malar, Amrita Daftary

**Affiliations:** 1 School of Global Health and Dahdaleh Institute of Global Health Research, York University, Toronto, Canada; 2 Department of African Studies and Linguistics, University of Cape Town, Cape Town, South Africa; 3 School of Health Policy & Management, York University, Toronto, Canada; 4 Independent Consultant, Nairobi, Kenya; 5 Migrant Clinicians Network, Austin, Texas, United States of America; 6 Stop TB Partnership, Geneva, Switzerland; 7 Centre for the AIDS Programme of Research in South Africa, University of KwaZulu-Natal, Durban, South Africa; PLOS: Public Library of Science, UNITED STATES

## Transforming the language used in tuberculosis care

Prior to COVID-19, tuberculosis (TB) was the world’s leading infectious disease challenge. Rates of incidence and mortality show no sign of decline, and people living in low- and middle-income countries and those facing social and material disadvantages remain disproportionately affected. The 2018 and upcoming 2023 United Nations High-Level Meetings on TB have galvanized political momentum and advocacy to coalesce efforts across all sectors, beyond health, and eliminate this ancient disease. Decision-makers have concurrently recognized the need to engage civil society so that elimination responses reflect allyship toward those who are directly affected [1]. Central to these burgeoning movements is the need to be more purposeful about the language used in describing and projecting ideas about TB, to trigger positive attention and stimulate transformative action [2].

A combination of speech and sound, language is a critical vehicle through which disease related discourses, and responses therein, become constructed and ingrained [3]. The literal, objective meaning underlying the words we use, and combinations thereof (phrases), are laden with implicit, contextual interpretations that are realized through our belief and value systems, and the environments in which those systems are shaped [4]. Regardless of intent, contextual meanings penetrate our subconscious and give way to discourse, a form of communication that is cyclically connected to a form of thinking that, over time, becomes normalized [3]. Normalized discourses support normalized practices, or responses that assume ‘natural’ status, shape ‘natural’ behaviour and become accepted without question. Events, circumstances, and people increasingly become associated with those discourses with the most dominant becoming normative, and fueling the social production of labels, stereotypes, and standards of worth, value, and acceptability, including for health-related issues [4]. Nowhere has this been more relevant than in the case of TB, a disease that is associated with poverty, deviance, and danger, that triggers deep social reactions–from disdain, horror, and culpability to sympathy and apathy, and is oft subjugated as a disease of the other [5].

TB stigma and discrimination are barriers to its elimination. The language used in TB-related communication has been implicated in the disenfranchisement stigmatization of people affected by TB leading, in instances, to violations of their human rights [6]. Technical medical jargon such as “case” (persons confirmed to have TB), “suspect” (person presumed to have, or under investigation for, TB), and “defaulter” (person who has stopped taking recommended treatments) feed into discourses of false blame and criminality [2, 7]. Acquisition of TB and poor responses to treatment are commonly framed as the responsibility of affected individuals rather than its biosocial determinants or arduous treatment course. Amidst this biomedical, normative and individualistic discourse, the political, financial and social underpinnings of TB are less easily engaged with.

Recent rights-based guidance about reimagining the language used in TB communication, ‘Words Matter: suggested language and usage for tuberculosis communications’, calls for the substitution of many stigmatizing terms [8]. Such laudable efforts, produced in partnership with and increasingly led by communities and persons with lived experience of TB, have potential to pivot public discourse and return social value to those who are affected by TB. As with other stigmatized conditions such as mental illness, substance use disorders, COVID-19, and HIV, recognizing the harms of poor language choices and recommending standards may help to restore the inclusion and respect of affected persons [9]. In TB, continuous reflection is needed to promote a discourse of inculpability and allyship with affected communities–including and beyond literal semantics–so that the disease is not only destigmatized but affected people unapologetically empowered. Notwithstanding overt stigmatizing terms, several other problematic references persist and need revisitation (it would be great to state why they need revision). Phrases that relegate TB and similar infections as diseases “of poverty” (pointing to the social and economic determinants) that are connected to “vulnerable” populations (alluding to persons at higher risk for acquiring or developing disease), including the “foreign-born” or “migrants” (denoting disease among newcomer populations [10]), are all too often used as catchall and without contextualization. While helping to emphasize social dimensions and call for wider investments, they can counter intentionally victimize communities, diminishing their dignity and innate resilience. Affected people may become painted as outsiders, inferior or weak, invoking benevolence and compassion but void of the allyship needed for their empowerment and self-determination [11]. This is evident in well-intentioned framings of women, racialized persons, and the elderly, where narratives of concern and compassion can capacitate their micro oppression and alienation [11, 12]. Emphasizing connections of TB to a particular country, race, ethnicity, gender, living condition, legal status, or income group, while crucial for health programming and resource allocation, could similarly reify its othering, especially if the opportunity to unveil and amplify the structural violence through which those connections are sustained is missed.

Amongst ongoing efforts in TB prevention, diagnostics, and treatment, the language and phraseology used to communicate risks about and invite actions on TB have a therapeutic role to play. The current age of open access to scientific information and public policy has rendered the language used within technocratic spaces to be widely available for public scrutiny and adoption. Indeed, the diffusion of stigmatizing language in settings beyond health, such as immigration, social welfare, labour, and law, have dissuaded people with TB from engaging with those services (for example, see Abarca Tomas, et al. 2013 [13]). Vitalized public interest and communication about infectious diseases in general, sparked by the COVID-19 pandemic, further reveal how language can fuel a particular discourse. Public framings about the origin of COVID-19 criminalized those who were first affected, and compounded inequities among those who were already marginalized [14]. Concurrently, outcry over unjust policies and political inactions world over provoked an unprecedented level of global connectedness. This birthed countervailing discourses of normality and inculpability about an infection that has remarkable similarity to TB in terms of its respiratory symptomology and airborne transmission.

In TB, community-led tools and actions denouncing its stigmatization and activating rights-based responses are increasingly emergent [15]. There is no time as the present to disrupt mainstay connotations and transform the words we use to talk about TB, so that affected persons are at the outset accepted and empowered.
